# Circulating matrix metalloproteinases are associated with arterial stiffness in patients with type 1 diabetes: pooled analysis of three cohort studies

**DOI:** 10.1186/s12933-017-0620-9

**Published:** 2017-10-25

**Authors:** Stijn A. Peeters, Lian Engelen, Jacqueline Buijs, Nish Chaturvedi, John H. Fuller, Anders Jorsal, Hans-Henrik Parving, Lise Tarnow, Simone Theilade, Peter Rossing, Casper G. Schalkwijk, Coen D. A. Stehouwer

**Affiliations:** 1 Department of Internal Medicine, Maastricht University Medical Centre, P.O. Box 5800, 6202 AZ Maastricht, The Netherlands; 2 Department of Internal Medicine, Zuyderland hospital, Heerlen, The Netherlands; 3 Centraal Bureau voor de Statistiek, Heerlen, The Netherlands; 4 Institute of Cardiovascular Sciences, University College London, London, UK; 5 Department of Epidemiology and Public Health, University College London, London, UK; 6 Steno Diabetes Center Copenhagen, Gentofte, Denmark; 7 Department of Cardiology, Aarhus University Hospital, Aarhus, Denmark; 8 Department of Clinical Medicine, Faculty of Health, Aarhus University, Aarhus, Denmark; 9 Department of Medical Endocrinology, Rigshospitalet, University of Copenhagen, Copenhagen, Denmark; 10 Faculty of Health, Aarhus University, Aarhus, Denmark; 11 University of Copenhagen, Copenhagen, Denmark; 12 CARIM School for Cardiovascular Diseases, Maastricht University Medical Centre, Maastricht, The Netherlands

**Keywords:** Type 1 diabetes, Matrix metalloproteinase, Tissue inhibitor of metalloproteinase, Pulse pressure, Arterial stiffness, Carotid-femoral pulse wave velocity

## Abstract

**Background:**

Altered regulation of extracellular matrix (ECM) composition by matrix metalloproteinases (MMPs) and tissue inhibitors of metalloproteinase (TIMPs) may contribute to arterial stiffening. We investigated associations between circulating MMP-1, -2, -3, -9, -10 and TIMP-1, and carotid-femoral pulse wave velocity (cfPWV) and pulse pressure (PP), as markers of arterial stiffness in type 1 diabetic patients.

**Methods:**

Individuals with type 1 diabetes from three different cohorts were included in this study: EURODIAB Prospective Complications study (n = 509), LEACE (n = 370) and PROFIL (n = 638). Linear regression analyses were used to investigate cross-sectional associations between circulating levels of MMP-1, -2, -3, -9, -10, and TIMP-1 and cfPWV (n = 614) as well as office PP (n = 1517). Data on 24-h brachial and 24-h central PP were available in 638 individuals from PROFIL. Analyses were adjusted for age, sex, duration of diabetes, HbA1c, mean arterial pressure (MAP), and eGFR, and additionally for other cardiovascular risk factors and presence of vascular complications.

**Results:**

After adjustment for potential confounders and presence of vascular complications, circulating MMP-3 was associated with cfPWV [β per 1 SD higher lnMMP3 0.29 m/s (0.02; 0.55)]. In addition, brachial and central 24-h PP measurements in PROFIL were significantly associated with MMP-2 [(1.40 (0.47:2.33) and 1.43 (0.63:2.23)]. Pooled data analysis showed significant associations of circulating levels of MMP-1 and MMP-2 with office PP [β per 1 SD higher lnMMP-1 and lnMMP-2 = − 0.83 mmHg (95% CI − 1.50; − 0.16) and = 1.33 mmHg (0.55; 2.10), respectively].

**Conclusions:**

MMPs-1, -2, and -3 are independently associated with markers of arterial stiffening in patients with type 1 diabetes and may become therapeutic targets.

**Electronic supplementary material:**

The online version of this article (doi:10.1186/s12933-017-0620-9) contains supplementary material, which is available to authorized users.

## Background

Arterial stiffening is an important pathway through which diabetes causes cardiovascular disease [1]. The current gold standard to quantify arterial stiffness [2] is carotid-femoral pulse wave velocity (cfPWV) measurements, which have shown to be significantly higher in type 1 diabetic patients with previous CVD compared to patients without CVD after multivariable adjustment [3]. In addition, previous studies in individuals with type 1 diabetes have shown independent associations between higher pulse pressure (PP), another and older marker of arterial stiffness, and incident cardiovascular disease (CVD) [4, 5] and all-cause mortality [4].

Although the pathogenic mechanism leading to greater arterial stiffness in type 1 diabetes has not been fully elucidated, extracellular matrix (ECM) remodelling, by matrix metalloproteinases (MMPs) and tissue inhibitor of metalloproteinases (TIMPs), is thought to contribute to alterations in vascular collagen and/or elastin content, proliferation and migration of vascular smooth muscle cells, and plaque instability, potentially leading to greater arterial stiffness, hypertension and subsequent micro- and macrovascular complications [6–10].

So far, studies reporting on associations between circulating levels of MMPs and TIMP on the one hand and arterial stiffness on the other have been inconsistent. cfPWV has been positively associated with (pro)MMP-1 [11–13], MMP-2 [14–16], MMP-9 [14] and TIMP-1 [17], whereas null associations between circulating levels of MMP-9 [15, 17, 18] and TIMP-1 [15, 18] and cfPWV have also been published. Only one study showed a positive association between MMP-2 and PP [19]. Most studies were relatively small and addressed only a limited number of MMPs, and none focused specifically on individuals with type 1 diabetes.

Therefore, the aim of our study was to investigate associations between circulating levels of MMP-1, -2, -3, -9, and -10 and of TIMP-1 with markers of arterial stiffness, i.e. cfPWV and PP, in individuals with type 1 diabetes.

## Methods

### Study population

To study possible associations between circulating markers of ECM remodelling and cfPWV, the current gold standard for arterial stiffness, we used the PROFIL study [3], which is a cross-sectional study that was conducted at the Steno Diabetes Center in Denmark, between 2009 and 2011, and included 676 individuals with type 1 diabetes. In the diabetes group, 316 patients were normoalbuminuric, 169 had microalbuminuria and 191 had macroalbuminuria (by design).

Further evaluation of arterial stiffness in individuals with type 1 diabetes, as expressed by PP, was performed by combining data from the PROFIL study with data from the EURODIAB Prospective Complications Study [20–22] and the LEACE study [23]. We added these analyses in multiple studies to investigate external validity and reproducibility, as cfPWV was only present in 1 study. In brief, the cross-sectional EURODIAB nested case–control study, which was performed as follow-up from the EURODIAB Prospective Complications Study, included 543 individuals with type 1 diabetes. Selected cases (n = 348) were those with one or more vascular complications, whereas controls (n = 195) were those without any evidence of vascular complications. The LEACE study is a prospective cohort study conducted at the Steno Diabetes Center in Denmark, with baseline measurements performed in 1993 when 391 individuals with type 1 diabetes were included. In this study group, 199 had diabetic nephropathy and 192 had normoalbuminuria (by design).

Individuals with missing data on MMPs or TIMP-1 (n = 25), office PP (n = 15) or potential confounders (n = 53) were excluded from the present analyses, resulting in a total study population of 1517 individuals (509 from EURODIAB, 370 from LEACE and 638 from PROFIL). The local Ethics Committees approved the studies and all patients gave informed consent.

### Main determinants

Concentrations of MMP-1, MMP-2, MMP-3, MMP-9, MMP-10 and TIMP-1 were determined in EDTA plasma (for LEACE) or serum samples (for EURODIAB and PROFIL) that were stored at − 80 °C until analyses. Circulating levels of MMPs and TIMP-1 were measured using a commercially available multi-array enzyme-linked immunosorbent assay (ELISA) kit [Human MMP-3-Plex Kit (for MMP-1, -3 and -9), Human MMP-2-Plex Kit (for MMP-2 and -10) and Human TIMP-1 Kit, MSD, Rockville, United States of America] according to the manufacturer’s protocol. The MMPs were detected in both a pro- and an active form. TIMP-1 was detected only in the active form. The intra- and inter-assay coefficients of variation were < 10% for MMP-1, -2, -3, -9, and -10 and TIMP-1 in all studies, except for the inter-assay coefficient of variation of MMP-1 in PROFIL (10.4%), the inter-assay coefficients of variation in all studies for MMP-3 (10.8–14.1%) and the inter-assay coefficient of variation for MMP-9 in LEACE (13.7%).

### Main outcomes

cfPWV and/or PP were studied as markers of arterial stiffness. cfPWV measurements were performed in the PROFIL study and were available in 614 of the 638 participants. Trained laboratory technicians performed the measurements with the SphygmoCor device (AtCor Medical, Sydney, Australia) in participants in a supine position after a resting period of 15 min. Three cfPWV measurements were recorded and the two measurements closest to each other were averaged and used in the analyses.

In the EURODIAB and the LEACE studies, a random zero sphygmomanometer (Hawskley, Lancing, UK) with the appropriate cuff size was used to measure arterial blood pressure. In the EURODIAB study, measurements were performed in a seated position after resting for 5 min [24], whereas in the LEACE study measurements were performed after at least 10 min of rest in supine position [25]. In the PROFIL study, brachial blood pressure was determined after resting for 15 min in supine position (A&D Medical, Tokyo, Japan) using an appropriate cuff size [26]. PP (mmHg) was calculated by subtracting diastolic blood pressure from systolic blood pressure. Besides PP, in 638 patients from the PROFIL study, data on 24-h ambulatory blood pressure measurements (ABPM) were available. A validated tonometric watch-like device (BPro, HealthStats, Singapore) was used to record these 24-h ABPM. This device captured radial pulse wave reflections and calculated ABPM [27, 28]. The device was calibrated to brachial blood pressure and calculated central blood pressure. Blood pressure was measured every 15 min. ABPM data were considered adequate when at least 14 and 7 measurements were performed during the day (07.00 a.m.–11.00 p.m.) and night (11.00 p.m.–07.00 a.m.), respectively, according to current guidelines [29]. A mean ± SD of 45 ± 7 successful measurements were recorded during the day and 18 ± 4 during the night.

### Potential confounders

Demographic data, information about medication, smoking history, duration of diabetes and use of antihypertensive medication were collected using questionnaires and patient records. Mean arterial pressure (MAP) was calculated as $\left[ {{\text{systolic blood pressure}} + \left( {2*{\text{diastolic blood pressure}}} \right)} \right]/3$. Body weight and height were measured to calculate the body mass index (BMI). Blood samples were taken for measurements of total cholesterol, glycated hemoglobin (HbA1c) and creatinine. We estimated the glomerular filtration rate (eGFR) using the Chronic Kidney Disease Epidemiology Collaboration (CKD-EPI) equation [30].

### Vascular complications

Vascular complications were defined as presence of macro- and/or microvascular complications, i.e. CVD, albuminuria or retinopathy. CVD was defined, in all studies, as a cardiovascular event in a patient’s medical history, including myocardial infarction, coronary intervention or stroke [3, 23, 31] and this definition is applicable to 96% of CVD in our total study population. In the EURODIAB study, angina and ischemic changes on ECG were also included in the definition of CVD [31]. In addition, (intervention for) peripheral arterial disease was additionally included in LEACE and PROFIL [3, 23].

Albumin excretion rates were measured in 24-h urine collections. Microalbuminuria was defined as albumin excretion rate (AER) between 20 and 200 µg/min or between 30 and 300 mg/24 h. Macroalbuminuria was defined as AER above 200 µg/min or 300 mg/24 h.

Retinopathy was assessed by retinal photography in all studies. Individuals were classified as no retinopathy, non-proliferative retinopathy, or proliferative retinopathy. In the PROFIL study blindness was also classified. Classification was conducted according to local protocols [3, 23, 32].

### Statistical analyses

All analyses were performed using the Statistical Package for Social Sciences (SPSS), version 20 (IBM Corporation, Armonk, NY, USA). Log-transformation was performed for variables with a skewed distribution (i.e. MMP-1, MMP-2, MMP-3, MMP-9, MMP-10 and TIMP-1).

One-way ANOVA and Chi square tests were performed for comparison of baseline characteristics between the tertiles of cfPWV and of office PP. Linear regression analyses were applied to examine associations between circulating levels of MMP-1, MMP-2, MMP-3, MMP-9, MMP-10 and TIMP-1 on the one hand and cfPWV (n = 614), office PP (n = 1517), or 24-h PP (n = 638) measurements on the other. Analyses of these associations were adjusted for age, sex, duration of diabetes, HbA1c, eGFR, MAP, cohort and double inclusion (model 1). One hundred and thirty four patients were included in both the LEACE and the PROFIL study. However, inclusion in PROFIL was more than 15 years later than in LEACE. Therefore we decided to adjust for this instead of deleting these cases, which could lead to selection bias. The latter two adjustments in model 1 were only performed in case of office PP analysis. Further adjustments were made for total cholesterol, BMI, smoking status, use of antihypertensive medication and presence of any vascular complication (model 2). Results of these analyses are presented as m/s higher cfPWV or mmHg higher PP per 1 SD higher level of circulating lnMMP or lnTIMP-1. For this purpose, the MMP and TIMP-1 Z-scores were calculated cohort-specifically as absolute values may not be comparable due to sample preparation (i.e., plasma in LEACE vs. serum in EURODIAB and PROFIL). Furthermore, analyses of the associations between MMP-1, -2, -3, -9, and -10 and the outcomes were adjusted for the MMP inhibitor TIMP-1, as an indication of MMP activity.

We additionally tested for interaction by variables that may modify the associations between MMPs and TIMP-1 on the one hand and cfPWV or PP on the other (i.e. sex or, in the case of analyses with office PP, cohort) by adding product terms between these variables and MMPs and TIMP-1 to the fully adjusted models. We stratified our analyses whenever significant interactions (p for interaction < 0.05) were found.

## Results

### Patient characteristics

Table 1 shows patient characteristics according to tertiles of cfPWV (n = 614). Higher cfPWV was significantly associated with higher age, male gender, higher BMI, longer duration of diabetes, higher systolic blood pressure, higher mean arterial pressure, higher PP and lower eGFR. In addition, higher cfPWV was positively associated with a higher prevalence of CVD, a higher degree of albuminuria and a higher degree of retinopathy. Circulating levels of MMP-1, MMP-2, MMP-3, MMP-9, MMP-10 and TIMP-1 were also significantly higher with higher cfPWV.

**Table 1:** Patient characteristics according to tertiles of cfPWV (n = 614)

	First tertile 4.5–8.5 m/s	Second tertile 8.6–11.3 m/s	Third tertile 11.4–23.4 mmHg	p value for linearity
	(n = 204)	(n = 205)	(n = 205)	
Age (years)	45.2 (12.3)	54.9 (9.4)	63.0 (8.6)	< 0.001
Sex (male/female, %)	51/49	53/47	62/38	0.047
BMI (kg/m^2^)	24.5 (3.5)	25.0 (4.2)	25.9 (4.0)	0.001
HbA1c (%)	7.9 (1.2)	8.2 (1.2)	8.0 (1.0)	0.054
HbA1c (mmol/mol)	63 (13.2)	66 (13.3)	64 (10.5)	0.054
Duration of diabetes (years)	20.9 (14.4)	33.8 (12.6)	42.5 (12.5)	< 0.001
Total cholesterol (mmol/l)	4.69 (0.78)	4.64 (0.90)	4.75 (0.88)	0.454
Smoking at baseline (%)	20.1	24.4	15.1	0.183
Systolic blood pressure (mmHg)	123 (12)	131 (16)	143 (18)	< 0.001
Diastolic blood pressure (mmHg)	74 (9)	76 (10)	74 (9)	0.145
Mean arterial pressure (mmHg)	90 (9)	94 (11)	97 (11)	< 0.001
Pulse pressure (mmHg)	49 (10)	55 (12)	69 (15)	< 0.001
cfPWV (m/s)	7.4 [6.5–7.8]	9.8 [9.1–10.5]	13.6 [12.3–15.5]	–
Antihypertensive medication (%)	43.1	77.6	89.8	< 0.001
eGFR (ml/min/1.73 m^2^)	100 [85–108]	89 [63–99]	74 [53–91]	< 0.001
Cardiovascular disease (%)	7.4	20.0	31.2	< 0.001
Albuminuria (normo-/micro-/macro-, %)	69/18/13	45/26/29	32/25/43	< 0.001
Retinopathy (no/non-proliferative/proliferative/blind, %)	44/41/15/0	14/52/32/2	5/34/56/5	< 0.001
Z-score lnMMP-1	− 0.23 (0.95)	0.11 (1.03)	0.08 (0.90)	< 0.001
Z-score lnMMP-2	− 0.47 (0.95)	− 0.02 (0.89)	0.43 (0.95)	< 0.001
Z-score lnMMP-3	− 0.37 (0.91)	0.00 (1.00)	0.38 (0.84)	< 0.001
Z-score lnMMP-9	− 0.18 (0.92)	0.11 (0.95)	0.07 (0.98)	0.004
Z-score lnMMP-10	− 0.27 (0.98)	0.07 (1.06)	0.18 (0.93)	< 0.001
Z-score lnTIMP-1	− 0.26 (0.74)	0.00 (1.28)	0.20 (0.85)	< 0.001

Data are presented as means (standard deviation), median [inter-quartile range], or percentages, as appropriate

*BMI* body mass index, *HbA1c* glycated hemoglobin, *eGFR* estimated glomerular filtration rate by CKD-EPI formula, *MMP* matrix metalloproteinase, *TIMP-1* tissue inhibitor of metalloproteinase-1

### Associations between MMPs, TIMP-1 and cfPWV

Table 2 shows the results of the analyses of the associations between MMPs, TIMP-1 and cfPWV in 614 participants from the PROFIL study. Circulating MMP-3 levels showed an independent association with cfPWV [0.29 m/s (0.02; 0.55)] after full adjustment for potential confounders and presence of vascular complications. Circulating levels of MMP-1, MMP-2, MMP-9, MMP-10 and TIMP-1 were not independently associated with cfPWV. As both age and duration of diabetes are significantly associated, and can thus lead to overadjustment, we reanalyzed the data without adjustment for diabetes duration. Results showed an independent association between MMP-2 and cfPWV, with an increase in cfPWV of 0.24 m/s (0.02; 0.46) per 1 SD higher lnMMP-2, after adjustment for the other potential confounders and presence of vascular complications. In addition, the association between MMP-3 and cfPWV became stronger in the fully adjusted model [0.38 (0.11; 0.46)]. The associations between MMP-1, MMP-9, MMP-10, and TIMP-1 with cfPWV did not materially change after adjustment for age instead of age and diabetes duration.

**Table 2:** Associations between serum levels of MMP-1, -2, -3, -9, and -10 and TIMP-1 and cfPWV (n = 614)

	Model	β	95% CI	p value
MMP-1	1	− 0.11	− 0.31; 0.08	0.241
	2	− 0.11	− 0.30; 0.08	0.263
	3	− 0.17	− 0.38; 0.03	0.098
MMP-2	1	0.05	− 0.16; 0.26	0.653
	2	0.06	− 0.16; 0.27	0.598
	3	0.07	− 0.15; 0.28	0.533
MMP-3	1	0.28	0.01; 0.54	0.041
	2	0.29	0.02; 0.55	0.037
	3	0.27	0.00; 0.54	0.047
MMP-9	1	0.09	− 0.10; 0.28	0.345
	2	0.09	− 0.11; 0.28	0.397
	3	0.06	− 0.14; 0.26	0.556
MMP-10	1	− 0.07	− 0.26; 0.13	0.493
	2	− 0.04	− 0.25; 0.16	0.673
	3	− 0.06	− 0.26; 0.14	0.568
TIMP-1	1	0.12	− 0.07; 0.32	0.211
	2	0.12	− 0.07; 0.32	0.212

β, standardized regression coefficient: indicates increase in cfPWV (in m/s) per 1 SD increase in lnMMPs and lnTIMP-1

*CI* confidence interval

Model 1: age, sex, duration of diabetes, HbA1c, eGFR, MAP

Model 2: model 1 + total cholesterol, BMI, smoking, use of antihypertensive medication, and presence of vascular complications

Model 3: model 2 + TIMP-1

### Associations between MMPs, TIMP-1 and PP

Additional file 1: Table S1 and S2 show the patient characteristics of the three studies separately and according to tertiles of PP, respectively. Table 3 shows the results of the pooled data analysis of associations between MMPs, TIMP-1 and office brachial PP. After adjustment for potential confounders, 1 SD higher MMP-1 was inversely associated with PP [− 0.83 (− 1.50; − 0.16)], whereas MMP-2 was positively associated with PP [1.33 (0.55; 2.10)] (Table 3, model 2). These associations did not materially change after additional adjustment for TIMP-1 (Table 3, model 3) or mutual adjustment for MMP-2 or MMP-1, respectively. MMP-3, MMP-9, MMP-10 and TIMP-1 were not independently associated with PP (Table 3). MMP-9 was only associated with PP after additional adjustment for TIMP-1 [− 0.71 (− 1.40; − 0.01)] (Table 3, model 3).

**Table 3:** Associations between circulating levels of MMP-1, -2, -3, -9, and -10 and TIMP-1 and PP (pooled data, n = 1517)

	Model	β	95% CI	p-value
MMP-1	1	− 0.81	− 1.48; − 0.15	0.017
	2	− 0.83	− 1.50; − 0.16	0.015
	3	− 1.08	− 1.81; − 0.35	0.004
MMP-2	1	1.31	0.55; 2.07	0.001
	2	1.33	0.55; 2.10	0.001
	3	1.32	0.55; 2.09	0.001
MMP-3	1	0.42	− 0.49; 1.32	0.366
	2	0.41	− 0.50; 1.31	0.378
	3	0.39	− 0.52; 1.30	0.401
MMP-9	1	− 0.55	− 1.20; 0.10	0.099
	2	− 0.59	− 1.25; 0.08	0.084
	3	− 0.71	− 1.40; − 0.01	0.047
MMP-10	1	− 0.31	− 1.01; 0.39	0.383
	2	− 0.37	− 1.09; 0.35	0.318
	3	− 0.39	− 1.11; 0.34	0.296
TIMP-1	1	0.21	− 0.51; 0.93	0.564
	2	0.21	− 0.53; 0.94	0.582

β, standardized regression coefficient: indicates increase in pulse pressure (in mmHg) per 1 SD increase in lnMMPs and lnTIMP-1

*CI* confidence interval

Model 1: age, sex, duration of diabetes, HbA1c, eGFR, MAP, cohort and double inclusion

Model 2: model 1 + total cholesterol, BMI, smoking, use of antihypertensive medication, and presence of vascular complications

Model 3: model 2 + TIMP-1

As brachial BP measurement may overestimate central SBP and PP more in younger individuals, we re-analyzed the data in individuals above 40 years of age (n = 974, pooled data) as arterial stiffness is higher at a younger age compared to non-diabetic individuals. After full adjustment for potential confounders, 1 SD higher MMP-1 was inversely associated with PP [− 1.53 (− 2.45; − 0.61)], whereas MMP-2 and MMP-3 were positively associated with PP [1.40 (0.45; 2.35)] and [1.59 (0.48; 2.70)], respectively. In addition, these three markers of ECM remodeling all remained independently associated with office PP when included in the analysis with all MMPs and TIMP-1 together.

In the PROFIL study only, in which data on 24-h brachial and 24-h central PP were available, only MMP-2 was significantly associated with 24-h brachial PP [1.40 (0.47; 2.33)] and 24-h central PP [1.43 (0.63; 2.23)] (Additional file 1: Table S3). MMP-2 remained significantly associated with 24-h PP measurement when only individuals above 40 were analyzed (data not shown).

### Additional analyses

Adjustment for CVD, albuminuria and retinopathy as separate variables, instead of combined into presence of any vascular complication, did not materially change the results (data not shown). Adjustment for ACE-inhibitors/angiotensin II receptor antagonists instead of antihypertensive therapy did also not materially change the results (data not shown). Additional adjustment, in individuals from the PROFIL study, for use of statins and heart rate did not materially change the associations either between MMPs and cfPWV/office PP or between MMP-2 and 24-h blood pressure measurements (data not shown).

The association between MMP-1 and PP in LEACE differed significantly from those in the other studies (p for interaction 0.015). MMP-1 was inversely associated with PP in EURODIAB [− 1.41 (− 2.59; − 0.23)] and non-significantly inversely in PROFIL [− 0.73 (− 1.64; 0.19)], whereas a non-significant positive association was observed in LEACE [0.20 (− 1.41; 1.81)] (Additional file 1: Table S4). No other significant interaction between MMPs/TIMP-1 and cohort was present with regard to the associations between MMPs, TIMP-1 and PP.

No significant sex-associated differences were observed in the associations between MMPs, TIMP-1 and cfPWV or PP (p for interaction, all > 0.05).

## Discussion

In the present study in individuals with type 1 diabetes, we showed that serum levels of MMP-3 were positively associated with cfPWV. In addition, circulating levels of MMP-1 were inversely, and of MMP-2 positively, associated with PP. These associations persisted after adjustment for potential confounders. Circulating levels of MMP-9, MMP-10, and TIMP-1 did not show associations with markers of arterial stiffening.

Our study is the first to show an independent association between serum MMP-3 and cfPWV in individuals with type 1 diabetes, after (extensive) adjustment for potential confounders. To the best of our knowledge, only two studies, in patients without type 1 diabetes, have investigated circulating MMP-3 levels and markers of arterial stiffness [33, 34]. Although none of these studies reported independent associations between circulating MMP-3 and cfPWV, 1-year treatment with perindopril, compared to placebo, was associated with a significant reduction in cfPWV as well as in plasma MMP-2 and plasma MMP-3 in 17 patients with Marfan Syndrome [33]. In addition, Sasamura et al. [34] observed a non-significant positive association between circulating MMP-3 and brachial ankle PWV (baPWV) in a small study of non-diabetic hypertensive patients.

MMP-3 may contribute to arterial stiffness by cleaving matrix collagens (type II, IV, IX, X), elastin, laminin and nidogen, leading to increased collagen turnover and decreased elastin content. MMP-3, similar to MMP-2, can also induce fibroblast-mediated matrix production by cleaving extracellular decorin and releasing TGF-β from the extracellular matrix [35]. In contrast to cfPWV, circulating levels of MMP-3 were not associated with office PP (pooled data) nor with 24-h PP measurements. This could be caused by the fact that MMP-3 mediated ECM remodeling might mainly be performed in descending part of the aorta and that its intrinsic actions in the ascending aorta and aortic arch might be limited due the difference in aortic tissue composition [36]. This could explain the different findings in the associations between MMP-3 on the one hand and cfPWV and PP measurements on the other. However, as office PP may overestimate central PP more in younger (healthy) individuals, as indicated by Laurent et al. [2], we showed that MMP-3 was also associated with office PP in individuals with type 1 diabetes above the age of 40, which is in line with the association between MMP-3 and cfPWV. In addition, Rönnback et al. [37] showed that similar PP levels were observed 15–20 years earlier in individuals with type 1 diabetes compared to non-diabetic controls. Moreover, in a prospective study in individuals with type 1 diabetes with a mean age of 39 years, brachial PP was positively associated with incident CVD [5].

Serum MMP-2 levels and cfPWV were not independently associated in our study, whereas circulating MMP-2 was associated with both office PP (pooled data) as well as 24-h PP measurements. Our result differs from studies performed in non-diabetic individuals, in whom positive associations between circulating levels of MMP-2 and cfPWV were observed [14–16]. However, our study is in accordance with the study by Coutinho et al. [19], in which a positive association between plasma MMP-2 and PP was observed in normotensive and hypertensive African-Americans in the GENOA study. These results were also supported by studies at tissue level [8, 38, 39]. Arteries of dialyzed chronic kidney disease patients compared to those of non-dialyzed chronic kidney disease patients and kidney donors [38] showed higher MMP-2 activity, which was associated with lower elastic fiber content and greater arterial stiffness. In addition, renal transplant recipients with diabetes had stiffer arteries compared to recipients without diabetes and controls, and this was significantly associated with higher MMP-2 and MMP-9 activity in the arterial wall [8]. Next, in aortic tissue of aging rats with higher systolic blood pressure, higher intimal and medial MMP-2 levels and MMP-2/TIMP-2 ratios were observed [39]. These increases were accompanied by greater vascular intima and media thickness as well as higher medial type III collagen and lower elastin content [39]. MMP-2 has the ability to release transforming growth factor β (TGF-β) from the ECM [35]. A TGF-β-induced increase in fibroblast-mediated ECM production thus may be a plausible mechanism to explain the strong association between MMP-2 and arterial stiffening [40].

Duration of diabetes appeared to be the major confounder in the association between MMP-2 and cfPWV and was significantly correlated with age. If duration of diabetes would not be taken into account, a positive association was in fact observed, which is in line with the association between MMP-2 and office PP as well as 24-h PP measurements. Therefore, the model with adjustment for both age and diabetes duration may have been overadjusted and our results are consistent with an important role for MMP-2 in pathophysiological vascular remodeling. However, we cannot exclude that both age and duration of diabetes were the major confounders in the association between MMP-2 and cfPWV.

We also showed that serum MMP-1 was borderline significantly inversely associated with cfPWV after adjustment for potential confounders and TIMP-1 levels and this is in contrast to two studies in non-diabetic hypertensive patients in whom (pro)MMP-1 was significantly and positively associated with cfPWV [11, 12]. Patients with diabetes were excluded in both studies and this may explain the different results. However, the inverse association between MMP-1 and cfPWV is in fact consistent with the inverse association between MMP-1 and PP (pooled data) that we observed. The difference in the associations between MMP-1 on the one hand and cfPWV and PP on the other might be caused by the differences in vascular wall content in the various parts of the aorta [36]. Circulating levels of MMP-1 were inversely associated with PP. To our knowledge, correlations between circulating (pro)MMP-1 and PP have only been published in studies that did not include individuals with type 1 diabetes [12, 41], and were not adjusted for potential confounders. In our crude analysis, 1SD higher lnMMP-1 was associated with a *higher* PP [1.36 (0.49; 2.23)]. However the association inverted after adjustment for potential confounders, with MAP being the most influential confounder. The fact that higher tissue MMP-1 levels have been associated with thoracic aortic aneurysm and thoracic aortic dissection [42] also indicates that higher MMP-1 levels may result in decreased thoracic aortic collagen content and/or concentration leading to decreased arterial stiffness. Nonetheless, evidence so far on the role of MMP-1 in collagen degradation and/or turnover and arterial stiffness is scarce and should be further investigated.

Published data on MMP-9 and PWV are contradictory. The lack of association between MMP-9 and cfPWV in the present study was in agreement with studies in patients with essential hypertension [17], coronary heart disease [18] and bicuspid aortic valves [15]. In contrast, Yasmin et al. [14] observed a positive and independent association between MMP-9 and cfPWV in both hypertensive patients and healthy individuals. In addition, serum MMP-9, but also MMP-3 and TIMP-1, was correlated with invasive aortic PWV measurements in patients with aortic stenosis [43]. However, the latter correlations were unadjusted for potential confounders. In contrast to all previous studies, an inverse association was observed serum MMP-9 and cfPWV in healthy subjects [44]. In accordance with our result, Coutinho et al. did also not observe an association between MMP-9, as well as TIMP-1, and PP [19]. Therefore, the role of MMP-9 in arterial stiffness remains unclear and warrants further investigation.

Our finding of a lack of association between TIMP-1 levels and cfPWV is supported by various other studies [15, 18, 34]. Only the study by Tan et al. [17] observed an independent association between TIMP-1 and cfPWV in hypertensive patients, but patients with macrovascular complications, diabetes and possible inflammatory diseases were excluded from this study.

Various studies investigated associations between circulating MMP-1 [45], MMP-2 [46, 47], MMP-3 [45], MMP-9 [46–49]. MMP-10 [45], and TIMP-1 [46] with other markers of arterial stiffness in both type 1 [48] and type 2 [45–47, 49] diabetic individuals, but did not find significant associations.

### Limitations

Several limitations are applicable to our study. First, we used PP as marker of arterial stiffness, but this only presents indirect information on arterial stiffness and it was measured under different circumstances in the three different studies. However, it has been shown to be an independent predictor of cardiovascular events and all-cause mortality in type 1 diabetic patients [1, 4, 5]. Second, although our study also included cfPWV measurements, the current gold standard for arterial stiffness, these were only available in one of the three included studies. Thirdly, we used circulating levels of MMPs and TIMP-1, because these can easily be obtained, but we do not know whether or not plasma levels truly reflect tissue levels or if they originate from one or more specific tissues. Finally, we cannot draw conclusions on the pathophysiological mechanism regarding the development of arterial stiffness, because of the cross-sectional design of our studies. In addition, the significant difference, between the LEACE and the other two studies, in the association between MMP-1 and PP may have been caused by the play of chance. Despite these limitations, our results showed largely consistent results throughout the three different studies with regard to the associations between MMP-1, MMP-2 and PP in individuals with type 1 diabetes.

## Conclusions

In individuals with type 1 diabetes, MMP-1 (inversely) and MMP-2 and -3 (both positively) were associated with markers of arterial stiffening (PP and cfPWV). The results suggest that better understanding of altered ECM remodeling in patients with type 1 diabetes could provide a potential therapeutic target, which could lead to decreased arterial stiffness and improved outcomes.

## Additional file

**Additional file 1: Table S1.** Baseline characteristics of the three studies. **Table S2.** Patient characteristics according to tertiles of PP (n=1517). **Table S3.** Associations between circulating levels of MMP-1, -2, -3, -9, and -10 and TIMP-1 and 24-h PP measurements in PROFIL. **Table S4.** Associations between circulating levels of MMP-1, -2, -3, -9, and -10 and TIMP-1 and PP per study.

## Abbreviations

CVD: cardiovascular disease
eGFR: estimated glomerular filtration rate
HbA1c: glycated hemoglobin A1c
MMP: matrix metalloproteinase
PP: pulse pressure
cfPWV: carotid-femoral pulse wave velocity
TIMP: tissue inhibitor of metalloproteinase
