# Supplementary material for: Circulating matrix metalloproteinases are associated with arterial stiffness in patients with type 1 diabetes: pooled analysis of three cohort studies
Source: Cardiovasc Diabetol. 2017 Oct 25;16:139. doi: 10.1186/s12933-017-0620-9 (PMC5657128; doi:10.1186/s12933-017-0620-9)
Supplement: Supplementary file 1 — Additional file 1: Table S1. Baseline characteristics of the three studies. Table S2. Patient characteristics according to tertiles of PP (n=1517). Table S3. Associations between circulating levels of MMP-1, -2, -3, -9, and -10 and TIMP-1 and 24-h PP measurements in PROFIL. Table S4. Associations between circulating levels of MMP-1, -2, -3, -9, and -10 and TIMP-1 and PP per study. [file 12933_2017_620_MOESM1_ESM.docx]

Table S1. Baseline characteristics of the three studies

|  |  |  |  | | EURODIAB | LEACE | PROFIL |
| --- | --- | --- | --- | --- | --- | --- | --- |
|  |  |  |  | | (n=509) | (n=370) | (n=638) |
| Age (years) |  |  |  | | 39.6 (10.0) | 41.8 (9.6) | 54.6 (12.6) |
| Sex (male/female, %) | |  |  | | 52/48 | 61/39 | 55/45 |
| BMI (kg/m^2^) |  |  |  | | 24.5 (3.2) | 23.9 (2.9) | 25.2 (4.0) |
| HbA1c (%) |  |  |  | | 8.5 (1.6) | 9.1 (1.4) | 8.0 (1.4) |
| HbA1c (mmol/mol) | | |  | | 70 (17.6) | 76 (15.8) | 64 (12.5) |
| Duration of diabetes (years) | | |  | | 21.5 (9.5) | 27.8 (8.0) | 32.6 (16.0) |
| Total cholesterol (mmol/l) | |  |  | | 5.31 (1.15) | 5.23 (1.19) | 4.69 (0.86) |
| Smoking at start of study (%) | | |  | | 31.0 | 46.5 | 20.8 |
| Systolic blood pressure  (mmHg) | | |  | | 123 (20) | 142 (23) | 132 (18) |
| Diastolic blood pressure  (mmHg) | | |  | | 75 (11) | 81 (13) | 74 (9) |
| Mean arterial pressure (mmHg) | |  |  | | 91 (12) | 102 (14) | 94 (11) |
| Pulse pressure (mmHg) | |  |  | | 48 (17) | 61 (18) | 58 (15) |
| Pulse wave velocity (m/s)  Antihypertensive medication (%) | | |  | | -  31.0 | -  43.2 | 9.8 [7.8-12.3]  71.0 |
| Statins (%) | |  |  | | - | - | 59.7 |
| eGFR (ml/min/1.73m^2^) | |  |  | | 102 [87-112] | 101 [75-113] | 88 [65-100] |
| Cardiovascular disease (%) | | |  | | 24.2 | 6.5 | 20.7 |
| Albuminuria (normo-/micro-/macro-, %) | | |  | | 61/16/23 | 46/0/54 | 47/24/29 |
| Retinopathy (no/non-proliferative/proliferative/blind, %) | | | | 45/26/29/0 | | 16/42/42/0 | 21/42/34/3 |
| MMP-1 (ng/ml) | |  |  | | 11.8 [6.7-19.0] | 3.2 [1.9-5.7] | 20.9 [13.0-30.6] |
| MMP-2 (ng/ml) | |  |  | | 106 [98-115] | 200 [173-236] | 142 [128-159] |
| MMP-3 (ng/ml) | |  |  | | 15.8 [9.9-24.6] | 15.1 [9.5-24.4] | 19.2 [12.8-28.8] |
| MMP-9 (ng/ml) | |  |  | | 118 [76-187] | 25.0 [15.7-44.6] | 161 [108-241] |
| MMP-10 (pg/ml) | |  |  | | 1207 [882-1881] | 723 [499-980] | 1167 [868-1671] |
| TIMP-1 (ng/ml) | |  |  | | 290 [238-338] | 179 [146-233] | 303 [262-353] |

| Data are presented as means (standard deviation), median [inter-quartile range], or percentages, as appropriate. | | |
| --- | --- | --- |
| BMI, body mass index; HbA1c, glycated hemoglobin; eGFR, estimated glomerular filtration rate by CKD-EPI formula; MMP, matrix metalloproteinase; TIMP-1, tissue inhibitor of metalloproteinase-1; blind*, blindness was present in patients in the PROFIL study |  |  |

Table S2. Patient characteristics according to tertiles of PP (n=1517)

|  |  |  |  | | First tertile  17-46 mmHg | Second tertile  47-59 mmHg | Third tertile  60-129 mmHg | p-value for linearity | |  |  |
| --- | --- | --- | --- | --- | --- | --- | --- | --- | --- | --- | --- |
|  |  |  |  | | (n=517) | (n=488) | (n=512) |  | |  |  |
| Age (years) |  |  |  | | 39.8 (10.8) | 46.3 (12.1) | 53.3 (12.6) | <0.001 | |  |  |
| Sex (male/female, %) | |  |  | | 52/48 | 57/43 | 57/43 | 0.229 | |  |  |
| BMI (kg/m^2^) |  |  |  | | 24.2 (3.1) | 24.7 (3.6) | 25.0 (3.8) | <0.001 | |  |  |
| HbA1c (%) |  |  |  | | 8.4 (1.5) | 8.3 (1.4) | 8.6 (1.5) | 0.046 | |  |  |
| HbA1c (mmol/mol) | | |  | | 69 (16.4) | 68 (14.8) | 71 (16.0) | 0.046 | |  |  |
| Duration of diabetes (years) | | |  | | 21.2 (10.7) | 27.3(12.3) | 34.8 (13.0) | <0.001 | |  |  |
| Total cholesterol (mmol/l) | |  |  | | 4.91 (0.99) | 4.99 (1.06) | 5.19 (1.19) | <0.001 | |  |  |
| Smoking at baseline (%) | | |  | | 30.6 | 33.0 | 28.1 | 0.248 | |  |  |
| Systolic blood pressure  (mmHg) | | |  | | 114 (12) | 128 (12) | 152 (17) | <0.001 | |  |  |
| Diastolic blood pressure  (mmHg) | | |  | | 76 (10) | 76 (12) | 77 (12) | 0.010 | |  |  |
| Mean arterial pressure (mmHg) | |  |  | | 88 (10) | 93 (12) | 102 (12) | <0.001 | |  |  |
| Pulse pressure (mmHg) | |  |  | | 39 (6) | 52 (4) | 75 (13) | - | |  |  |
| Antihypertensive medication (%) | | |  | | 29.8 | 50.8 | 71.1 | <0.001 | |  |  |
| eGFR (ml/min/1.73m^2^) | |  |  | | 104 [90-114] | 98 [79-108] | 83 [59-99] | <0.001 | |  |  |
| Cardiovascular disease (%) | | |  | | 14.9 | 16.2 | 24.0 | <0.001 | |  |  |
| Albuminuria (normo-/micro-/macro-, %) | | |  | | 68/13/19 | 51/16/33 | 37/17/46 | <0.001 | |  |  |
| Retinopathy (no/non-proliferative/proliferative/blind*, %) | | | | 44/33/22/1 | | 26/38/35/1 | 13/38/47/2 | <0.001 | |  |  |
| Z-score lnMMP-1 | |  |  | | -0.06 (0.97) | 0.05 (1.04) | 0.01 (0.99) | 0.288 | |  |  |
| Z-score lnMMP-2 | |  |  | | -0.47 (0.80) | 0.00 (0.89) | 0.48 (1.05) | <0.001 | |  |  |
| Z-score lnMMP-3 | |  |  | | -0.19 (0.96) | -0.07 (1.02) | 0.26 (0.97) | <0.001 | |  |  |
| Z-score lnMMP-9 | |  |  | | 0.07 (0.95) | -0.01 (1.01) | -0.06 (1.04) | 0.045 | |  |  |
| Z-score lnMMP-10 | |  |  | | -0.01 (0.99) | 0.02 (1.01) | -0.01 (1.00) | 0.985 | |  |  |
| Z-score lnTIMP-1 | |  |  | | -0.08 (1.00) | -0.04 (1.09) | 0.12 (0.90) | 0.002 | |  |  |
| Data are presented as means (standard deviation), median [inter-quartile range], or percentages, as appropriate. | | | | | | | | | | | |
| BMI, body mass index; HbA1c, glycated hemoglobin; eGFR, estimated glomerular filtration rate by CKD-EPI formula; MMP, matrix metalloproteinase; TIMP-1, tissue inhibitor of metalloproteinase-1; *a category for blindness was only included in the PROFIL study | | | | | | | | |  |  |  |

Table S3. Associations between circulating levels of MMP-1,

-2, -3, -9, and -10 and TIMP-1 and 24-h PP measurements in PROFIL

|  |  |  |  | PROFIL  brachial 24h  (n=638) |  |  | PROFIL  central 24h  (n=638) |  |
| --- | --- | --- | --- | --- | --- | --- | --- | --- |
|  | Model |  | β | 95% CI | p-value | β | 95% CI | p-value |
| MMP-1 | 1 |  | 0.01 | -0.81;0.83 | 0.986 | -0.02 | -0.73;0.69 | 0.953 |
|  | 2 |  | 0.04 | -0.78;0.86 | 0.924 | 0.02 | -0.69;0.73 | 0.960 |
|  | 3 |  | -0.08 | -0.96;0.80 | 0.854 | -0.04 | -0.80;0.72 | 0.912 |
| MMP-2 | 1 |  | **1.48** | **0.56;2.40** | **0.002** | **1.51** | **0.71;2.31** | **<0.001** |
|  | 2 |  | **1.40** | **0.47;2.33** | **0.003** | **1.43** | **0.63;2.23** | **<0.001** |
|  | 3 |  | **1.43** | **0.50;2.36** | **0.003** | **1.45** | **0.65;2.25** | **<0.001** |
| MMP-3 | 1 |  | 0.26 | -0.81;1.34 | 0.631 | 0.22 | -0.72;1.15 | 0.651 |
|  | 2 |  | 0.27 | -0.81;1.35 | 0.620 | 0.24 | -0.70;1.17 | 0.619 |
|  | 3 |  | 0.24 | -0.84;1.33 | 0.659 | 0.22 | -0.72;1.16 | 0.641 |
| MMP-9 | 1 |  | -0.12 | -0.92;0.68 | 0.772 | -0.22 | -0.92;0.47 | 0.533 |
|  | 2 |  | -0.02 | -0.84;0.80 | 0.959 | -0.11 | -0.83;0.60 | 0.756 |
|  | 3 |  | -0.09 | -0.93;0.75 | 0.829 | -0.15 | -0.88;0.58 | 0.681 |
| MMP-10 | 1 |  | 0.18 | -0.69;1.04 | 0.687 | 0.15 | -0.60;0.90 | 0.695 |
|  | 2 |  | 0.13 | -0.77;1.02 | 0.780 | 0.16 | -0.61;0.94 | 0.678 |
|  | 3 |  | 0.09 | -0.81;0.99 | 0.839 | 0.15 | -0.63;0.93 | 0.710 |
| TIMP-1 | 1 |  | 0.20 | -0.66;1.06 | 0.646 | 0.03 | -0.72;0.77 | 0.941 |
|  | 2 |  | 0.34 | -0.53;1.20 | 0.444 | 0.17 | -0.58;0.92 | 0.660 |

β, standardized regression coefficient: indicates increase in pulse pressure (in mmHg) per

1 SD increase in lnMMPs and lnTIMP-1. CI: confidence interval.

Model 1: age, sex, duration of diabetes, HbA1c, eGFR, MAP

Model 2: model 1 + total cholesterol, BMI, smoking, use of antihypertensive medication, and presence of vascular complications

Model 3: model 2 + TIMP-1

Table S4. Associations between circulating levels of MMP-1, -2, -3, -9, and -10 and

TIMP-1 and PP per study

|  |  |  | EURODIAB  (n=509) |  |  |  | LEACE  (n=370) |  |  | PROFIL  (n=638) |  |
| --- | --- | --- | --- | --- | --- | --- | --- | --- | --- | --- | --- |
|  | Model | β | 95% CI | p-value |  | β | 95% CI | p-value | β | 95% CI | p-value |
| MMP-1 | 1 | **-1.44** | **-2.60;-0.29** | **0.015** |  | 0.31 | -1.25;1.87 | 0.693 | -0.77 | -1.68;0.15 | 0.100 |
|  | 2 | **-1.41** | **-2.59;-0.23** | **0.019** |  | 0.20 | -1.41;1.81 | 0.808 | -0.73 | -1.64;0.19 | 0.118 |
|  | 3 | **-2.14** | **-3.50;-0.77** | **0.002** |  | -0.04 | -1.73;1.65 | 0.964 | -0.82 | -1.80;0.16 | 0.100 |
| MMP-2 | 1 | 1.26 | -0.05;2.57 | 0.060 |  | 1.47 | -0.50;3.44 | 0.144 | **1.35** | **0.32;2.37** | **0.010** |
|  | 2 | **1.36** | **0.03;2.70** | **0.046** |  | 1.41 | -0.63;3.45 | 0.176 | **1.31** | **0.27;2.35** | **0.013** |
|  | 3 | **1.34** | **0.01;2.68** | **0.049** |  | 1.35 | -0.70;3.40 | 0.195 | **1.31** | **0.27;2.36** | **0.013** |
| MMP-3 | 1 | -0.27 | -1.91;1.37 | 0.746 |  | -0.22 | -2.30;1.85 | 0.833 | **1.24** | **0.05;2.44** | **0.041** |
|  | 2 | -0.23 | -1.89;1.42 | 0.783 |  | -0.27 | -2.36;1.82 | 0.799 | **1.25** | **0.05;2.45** | **0.042** |
|  | 3 | -0.26 | -1.91;1.40 | 0.763 |  | -0.43 | -2.55;1.68 | 0.688 | **1.26** | **0.05;2.46** | **0.041** |
| MMP-9 | 1 | **-1.00** | **-2.15;0.15** | **0.087** |  | 0.08 | -1.38;1.54 | 0.911 | -0.29 | -1.18;0.60 | 0.523 |
|  | 2 | **-1.04** | **-2.20;0.13** | **0.081** |  | -0.14 | -1.76;1.48 | 0.866 | -0.24 | -1.15;0.68 | 0.613 |
|  | 3 | **-1.57** | **-2.89;-0.24** | **0.021** |  | -0.26 | -1.90;1.38 | 0.753 | -0.24 | -1.18;0.70 | 0.613 |
| MMP-10 | 1 | -0.17 | -1.40;1.05 | 0.780 |  | -1.27 | -2.84;0.30 | 0.111 | 0.42 | -0.54;1.38 | 0.392 |
|  | 2 | -0.18 | -1.47;1.11 | 0.782 |  | -1.39 | -3.01;0.24 | 0.094 | 0.47 | -0.53;1.47 | 0.356 |
|  | 3 | -0.21 | -1.50;1.08 | 0.747 |  | -1.40 | -3.03;0.22 | 0.090 | 0.48 | -0.53;1.48 | 0.351 |
| TIMP-1 | 1 | 0.39 | -0.84;1.63 | 0.534 |  | 1.06 | -0.80;2.92 | 0.263 | -0.12 | -1.08;0.83 | 0.803 |
|  | 2 | 0.37 | -0.88;1.62 | 0.563 |  | 0.92 | -1.01;2.86 | 0.349 | -0.03 | -0.99;0.94 | 0.957 |

β, standardized regression coefficient: indicates increase in pulse pressure (in mmHg) per 1 SD increase in lnMMPs

and lnTIMP-1. CI: confidence interval.

Model 1: age, sex, duration of diabetes, HbA1c, eGFR, and MAP

Model 2: model 1 + total cholesterol, BMI, smoking, use of antihypertensive medication, and presence of vascular complications

Model 3: model 2 + TIMP-1
